# Matrix metalloproteinase-2 Promotes αvβ3 Integrin-Mediated Adhesion and Migration of Human Melanoma Cells by Cleaving Fibronectin

**DOI:** 10.1371/journal.pone.0041591

**Published:** 2012-07-27

**Authors:** Yang Jiao, Xue Feng, Yinpeng Zhan, Ruifei Wang, Sheng Zheng, Wenguang Liu, Xianlu Zeng

**Affiliations:** Institute of Genetics and Cytology, Northeast Normal University, Changchun, Jilin, China; Ecole Polytechnique Federale de Lausanne, Switzerland

## Abstract

**Background:**

Matrix metalloproteinase-2 (MMP-2) is a key regulator in the migration of tumor cells. αvβ3 integrin has been reported to play a critical role in cell adhesion and regulate the migration of tumor cells by promoting MMP-2 activation. However, little is known about the effects of MMP-2 on αvβ3 integrin activity and αvβ3 integrin-mediated adhesion and migration of tumor cells.

**Methodology/Principal Findings:**

Human melanoma cells were seeded using an agarose drop model and/or subjected to in vitro analysis using immunofluorescence, adhesion, migration and invasion assays to investigate the relationship between active MMP-2 and αvβ3 integrin during the adhesion and migration of the tumor cells. We found that MMP-2 was localized at the leading edge of spreading cells before αvβ3 integrin. αvβ3 integrin-mediated adhesion and migration of the tumor cells were inhibited by a MMP-2 inhibitor. MMP-2 cleaved fibronectin into small fragments, which promoted the adhesion and migration of the tumor cells.

**Conclusion/Significance:**

MMP-2 cleaves fibronectin into small fragments to enhance the adhesion and migration of human melanoma cells mediated by αvβ3 integrin. These results indicate that MMP-2 may guide the direction of the tumor cell migration.

## Introduction

Tumor metastasis characterized by the dissemination of tumor cells from a primary site to the distant sites is the most frequent cause of death for cancer patients [1], [2]. The initial step of tumor metastasis is a process of invasive tumor cell migrating in basement membrane, which implicates cell adhesion and migration as well as proteolysis of the extracellular matrix (ECM). This step involves many molecules including matrix metalloproteinases (MMPs) and integrins [3]–[16].

MMPs are a family of zinc-dependent matrix-degrading enzymes, which include collagenases, stromelysins, and gelatinases. MMPs regulate various cell behaviors through their basic function in protein degradation [10]–[23]. The basement membrane, the first barrier for the invading epithelial tumor cells, is mainly composed of type IV collagen and fibronectin, which are degraded primarily by type IV collagenases and gelatinases[11]–[15], [20], [22], [24]–[26]. MMP-2 (72 kDa) and MMP-9 (92 kDa) are two gelatinases.

Integrins are heterodimeric adhesion molecules, composed of noncovalently associated transmembrane glycoproteins α and β units, which connect adhesive proteins in the ECM to the intracellular actin cytoskeleton [27]–[33]. Integrins exist in different states of activation which determine the avidity and affinity of integrins to their ligands [4], [30]–[34]. αvβ3 integrin is a receptor of proteins with an exposed Arg-Gly-Asp (RGD) tripeptide, including vitronectin, fibronectin, fibrinogen, thrombospondin, osteopontin, von Willebrand factor, and some degraded laminins and collagens [27], [31], [35], [36]. Numerous studies have documented the marked differences in the surface expression and distribution of integrins in malignant tumor cells [37]. αvβ3 integrin is expressed strongly on the surface of malignant melanoma cells and angiogenic blood vessels, but weakly on pre-neoplastic melanomas and quiescent blood vessels [30], [38]. Furthermore, inducing the expression of the integrin subunit αv or β3 increased the metastatic potential of a melanoma cell line [19], [28], [39], [40].

MMP-2 directly binds to αvβ3 integrin, which is a regulator of MMP-2 activation during tumor cell migration [15], [41]–[43]. However, whether MMP-2, in turn, contributes to αvβ3 integrin-mediated tumor cell migration is not clear. We examined the expression of MMP-2 and αvβ3 integrin in human A375 melanoma cells and human M21 melanoma cells using immunofluorescence staining, and demonstrated that MMP-2 accumulated at the leading edge of migrating cells before αvβ3 integrin. Given these findings, we addressed whether MMP-2 was an important regulator of αvβ3 integrin-mediated melanoma cell migration. Our results showed that inhibition of MMP-2 activity in the tumor cells dramatically decreased the adhesion and migration of the tumor cells.

## Materials and Methods

### Cell Culture and Proteinase Inhibitors

Human A375 melanoma cells were purchased from the Cell Bank of Type Culture Collection of the Chinese Academy of Science (Shanghai, China). Human melanoma cell M21 and M21-L were from the School of Basic Medical Sciences, Jilin University of China, and the M21-L is a mutant cell line lacking αvβ3 integrin [44]. The cells were grown at 37°C in a humidified atmosphere with 5% CO_2_ in IMDM containing 10% fetal bovine serum (FBS). Serum starvation of cultures was performed as follows: cells were cultured in serum-free medium for 24 h to synchronize the cell cycle at the G_0_ stage. GM6001 (Chemicon, Temecula, CA) is a broad spectrum hydroxamate MMP inhibitor, and its IC_50_ values have been reported as follows: 0.4 nM for MMP-1; 0.5 nM for MMP-2; 27 nM for MMP-3; 0.1 nM for MMP-8 and 0.2 nM for MMP-9 [45].

### Statistics of the Cell Morphology

Cells in the logarithmic phase were trypsinized, re-suspended in IMDM containing 10% FBS, and incubated with MMPs inhibitor (GM6001) at different concentrations (0.4 nM, 0.5 nM, 27 nM, 0.1 nM and 0.2 nM) or with arginine-glycine-aspartate RGD peptides (Sigma, 0.1 mg/ml). The treated cells were seeded in 6-well plates coated with human fibronectin and cultured at 37°C in a humidified atmosphere with 5% CO_2_. The cell morphology was observed at different time points (3 h, 6 h, 9 h, 12 h, 15 h, and 18 h) using microscopy.

### Assay of Cell Migration in the Agarose Drop

Cell migration was quantified by measuring the extent of cell migrating out of the agarose drops using a modified method [46]. After 24 h of serum starvation, cells were trypsinized and re-suspended at a density of 5×10^6^ cells/ml in serum-free IMDM containing 0.3% low melting-point agarose (Invitrogen), and the mixture was maintained at 37°C to prevent the setting of agarose. Two-microliter drops were seeded on fibronectin-coated coverslips in a 24-well plate and then placed at 4°C for 20 min to allow the agarose getting solid. Different concentration of GM6001 was used in this model. Cell migration was measured at 24 h intervals during 3 days under an inverted microscope. The drop area and total area (area of the drop + area of migrating cells) were measured, and cell migration was expressed as the extent of the drop = [(total area/drop area)×100] - 100. The mean values ± SEM were obtained from at least three independent experiments.

### Transwell Assay

After 24 h of serum starvation, cells were trypsinized and re-suspended in serum-free IMDM at a density of 1×10^5^ cells/ml. The cells were seeded onto the top of the insert that was previously coated with fibronectin in serum-free medium containing different agent. Serum was placed in the lower chamber and the cultures were maintained at 37°C in a humidified atmosphere with 5% CO_2_.

### Immunofluorescence Assay in the Agarose Drop Model

Cells were cultured in the agarose drops on coverslips. The coverslips were fixed in 4% paraformaldehyde and rinsed with phosphate buffer saline (PBS). The cells were pre-incubated with 5% FBS for 30 min and incubated with the following primary antibodies: mouse anti-human αvβ3 (1/100, Millipore) and rabbit anti-human MMP-2 (1/100, Santa Cruz, CA). The antibodies were diluted in PBS containing 5% FBS for 1 h before use. The primary antibody-labeled cells were rinsed with PBS, and then incubated with anti-mouse TRITC-conjugated or anti-rabbit FITC-conjugated secondary antibody for 30 min, and then rinsed with PBS. The coverslips were mounted and observed under a confocal microscope (Olympus, Japan). Images were analyzed using the Olympus confocal software.

### Cell Adhesion Experiment

Forty-eight-well plates were coated with 10 µg/ml human fibronectin for 20 min at 37°C. Before addition of cells, the wells were blocked with 0.5% bovine serum albumin (BSA) for 30 min to prevent nonspecific binding. Cells were trypsinized and incubated with calcein AM (It can be transported through the cellular membrane into live cells, which makes it useful for testing cell viability and for short-term labeling of cells) at 37°C for 30 min. The cells were seeded on fibronectin at a density of 1×10^6^ cells/ml in serum-free medium, alone or with different concentration of GM6001, for 15 min at 37°C. The adhesion assay was halted by adding a large volume of PBS, and the loosely attached cells were washed off. Fluorescence detection of calcein AM in the plates was conducted using the fluorescence image plate reader (American Molecular Devices. Inc. Gemini EM) with excitation 485 nm and emission 535 nm.

### MMP-2 Cleavage of Fibronectin

Recombinant human MMP-2 (R&D Systems) was activated using p-aminophenylmercuric acetate (APMA) (Merck) for 1 h at 37°C. Full-length fibronectin was cleaved by activated MMP-2 for 2 h at 37°C. One hundred nanograms of MMP-2 and APMA-activated MMP-2 was analyzed by gelatin zymography. MMP-2 cleavage of fibronectin was analyzed using a 10% Tris-HCl gel by silver staining. For the nitrocellulose adhesion assay [47], full-length or MMP-2 cleaved fibronectin was resolved on a native Tris-HCl gel (4%–20%). Proteins were transferred on nitrocellulose, and the blot was washed with PBS and incubated with A375 cells for 4 h. After adhesion, the blot was washed, cells were fixed with 10% formaldehyde, stained with 0.1% amido black, and destained with a methanol/acetic acid/water solution (90:2:8). Cells bound to nitrocellulose was shown as dark blue.

**Figure 1:**
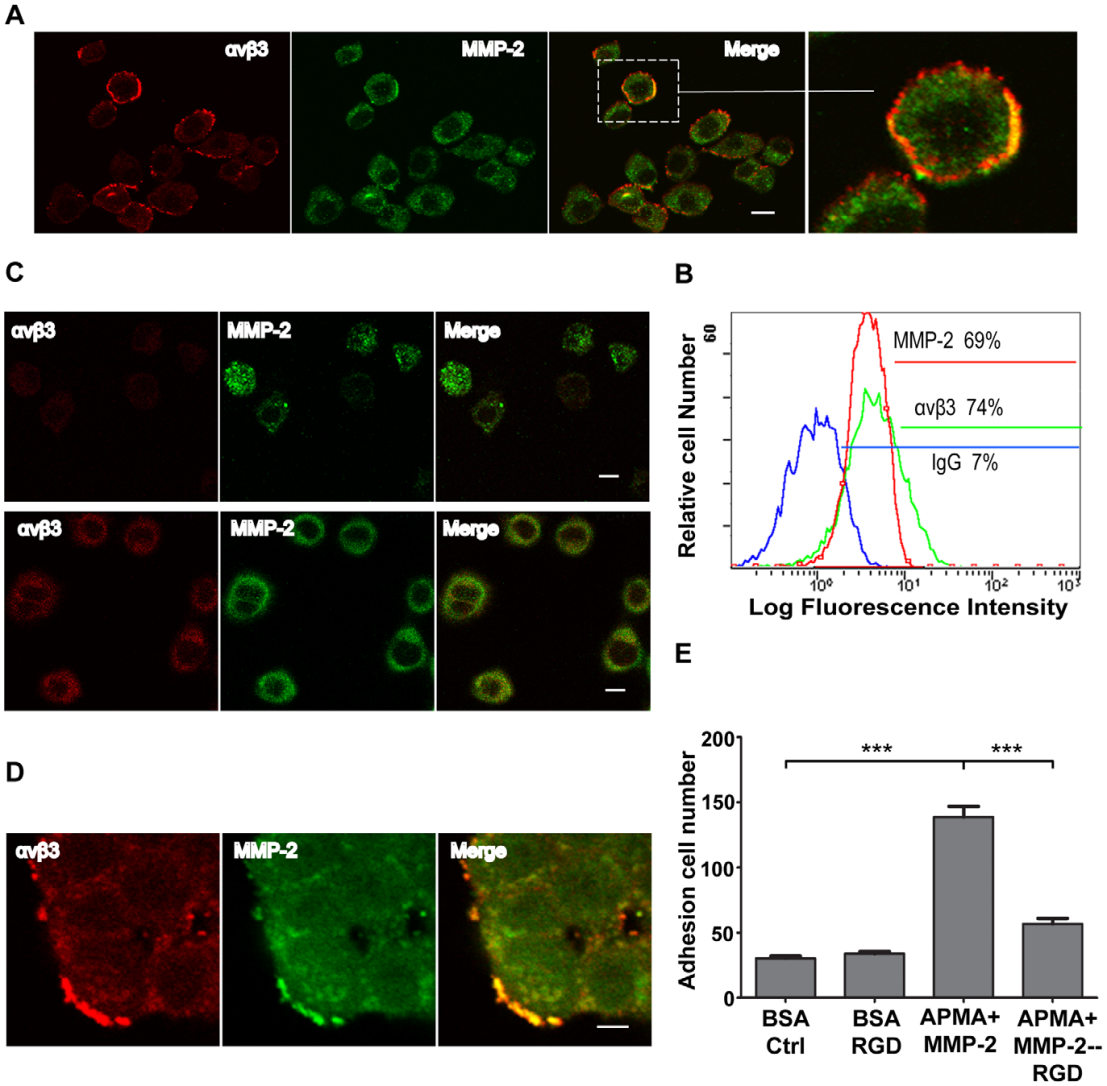
Expression of MMP-2 and αvβ3 in human A375 melanoma cells. (A) Cells were suspended in PBS and labeled with anti-αvβ3 (red) and anti-MMP-2 (green) antibodies. Scale bar = 10 µm. (B) Flow cytometric analysis of αvβ3 integrin and MMP-2 expression on the cell surface. The expression of αvβ3 integrin and MMP-2 was evaluated using anti-αvβ3 and anti-MMP-2 antibodies, and isotype IgG was used as a negative control. (C) A375 cells were seeded onto coverslips coated with human fibronectin for 30 min (upper panel) and 3 h (lower panel), then labeled with anti-αvβ3 (red) and anti-MMP-2 (green) antibodies. Scale bar = 10 µm. (D) A375 cells in agarose drops were seeded onto coverslips coated with human fibronectin. The cells were labeled with anti-αvβ3 (green) and anti-MMP-2 (red) antibodies 24 h after seeding and observed under a confocal microscope. Scale bar = 10 µm. (E) A375 cells were fluorescently stained and seeded on 96 well flat-bottom plates coated with APMA-activated recombinant human MMP-2 or 0.5% BSA. The control cells were treated using RGD peptides.

**Figure 2:**
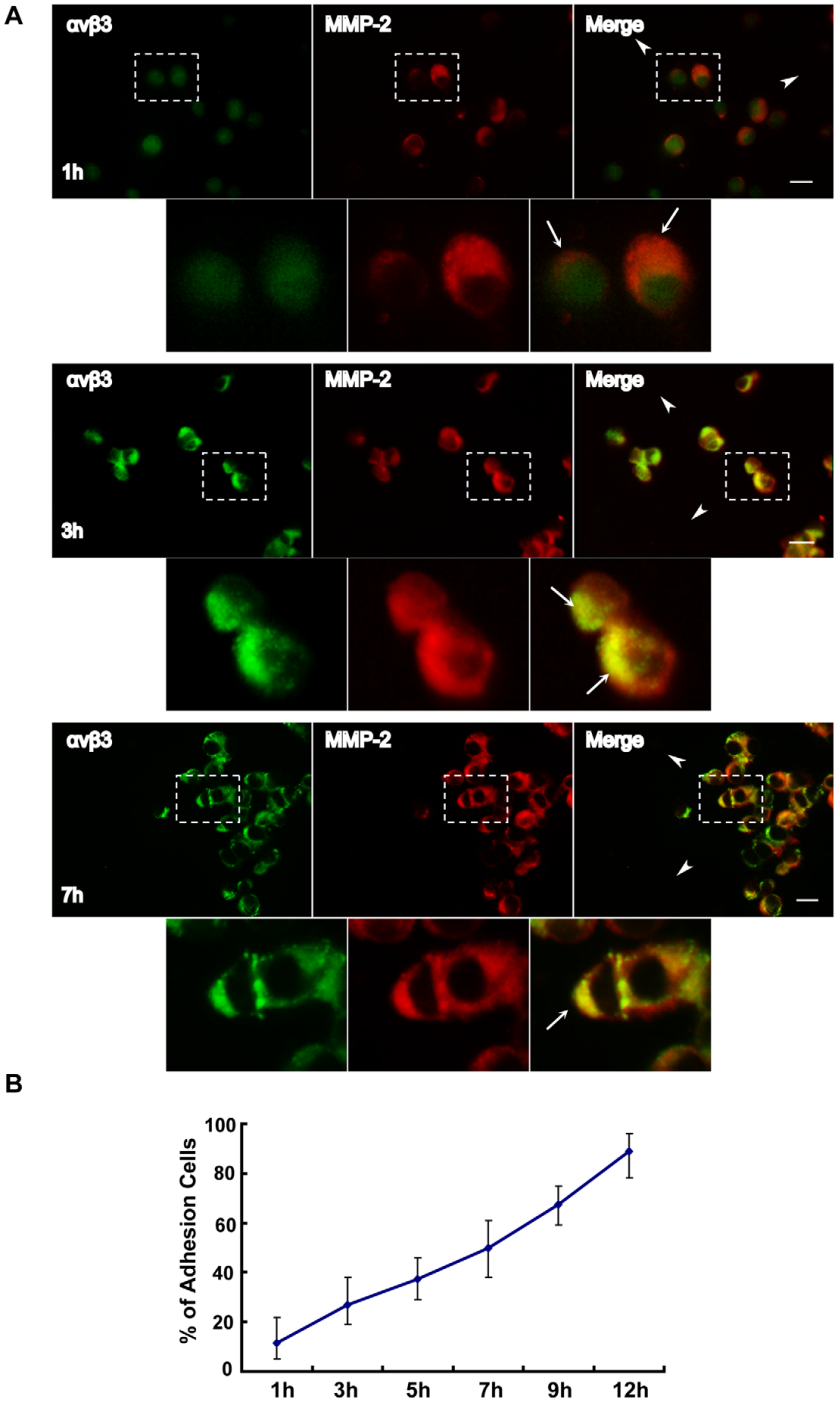
Distribution of MMP-2 and αvβ3 integrin in migrating human A375 melanoma cells. (A) human A375 melanoma cells in agarose drops were seeded onto coverslips coated with human fibronectin. The cells were labeled with anti-αvβ3 (green) and anti-MMP-2 (red) antibodies and observed under a confocal microscope at 1 h, 3 h or 7 h after seeding. The second row of each panel represents the blowups of the first row at the designated regions. The shorter arrows indicate the direction of cell migration, and the longer arrows indicate the co-localization of MMP-2 and αvβ3 integrin. Scale bar = 10 µm. (B) The percentages of adherent cells were calculated at different time points (1 h, 3 h, 5 h, 7 h, 9 h, 12 h).

## Results

### MMP-2 and αvβ3 Integrin are Expressed in Human A375 Melanoma Cells

To study the relationship of MMP-2 and αvβ3 integrin in tumor cell adhesion and migration, human A375 melanoma cells were suspended or seeded onto coverslips coated with human fibronectin. The expression of MMP-2 and αvβ3 integrin on the tumor cells was evaluated by immunofluorescence method. As shown in Figure 1, MMP-2 and αvβ3 integrin were expressed on the surface of suspended A375 cells and partially co-localized with each other (Figure 1A). The flow cytometric analysis (FACS) also showed the expression of MMP-2 and αvβ3 integrin on the cell surface (Figure 1B). We further investigated the co-localization of MMP-2 and αvβ3 integrin during cell spreading. The fluorescence signal for MMP-2 was detectable on the cell surface 30 min after the cells were seeded onto the coverslip (Figure 1C upper panel). However, the signal of co-localization between MMP-2 and αvβ3 integrin was very weak during this time. At 3 h after seeding (Figure 1C lower panel), the cells partially spread out, and the fluorescence signals of MMP-2 and αvβ3 integrin appeared stronger on the cell surface and they were partly co-localized. An agarose drop model was used to detect the co-localization of MMP-2 and αvβ3 integrin in the migrating cells (Figure 1D). After 24 h, both MMP-2 and αvβ3 integrin were strongly expressed on the surface of cells and partially co-localized with each other. A static adhesion assay was employed to detect the association between MMP-2 and αvβ3 integrin on A375 cells. As shown in Figure1 E, approximately more than 59% tumor cells adhered to the culture wells coated with APMA-activated MMP-2 compared with those cells blocked with RGD peptides. These results demonstrated that MMP-2 and αvβ3 integrin were expressed on A375 cells, and they might interact with each other.

### MMP-2 is Recruited to the Leading Edge of Invasive Human A375 Melanoma Cells before αvβ3 Integrin during Migration

To further study the interaction between MMP-2 and αvβ3 integrin, we cultured the human A375 melanoma cells using an agarose drop model with the following steps: 2 µl drops of cells (5×10^6^ cells/ml) in the agarose were seeded onto fibronectin-coated coverslips in a 24-well plate. The agarose drop model was used to demonstrate the direction of cell migration by forcing cells to move from a high cell density area to a low cell density area. The coverslips were then examined by using immunofluorescence method at different time points. As shown in Figure 2A and 2B, approximately 12% of cells adhered to the fibronectin-coated coverslip at 1 h after seeding. αvβ3 integrin was evenly distributed on the cells and the cluster-like signal of MMP-2 appeared at the invasive leading edge of the cells (Figure 2A, upper panel). Co-localization of the two molecules was not obviously observed on this time point. At 3 h after seeding (Figure 2A, middle panel), approximately 23% of cells adhered to the coverslip, and a partial co-localization of MMP-2 and αvβ3 integrin at the invasive front of the cells was observed. The co-localization of MMP-2 and αvβ3 integrin in a large number of adherent cells (approximately 47%) was observed at 7 h after seeding (Figure 2A, lower panel). The results indicated that MMP-2 was recruited to the leading edge of invasive tumor cells before αvβ3 integrin during migration, implying that MMP-2 may regulate αvβ3 integrin recruitment.

**Figure 3:**
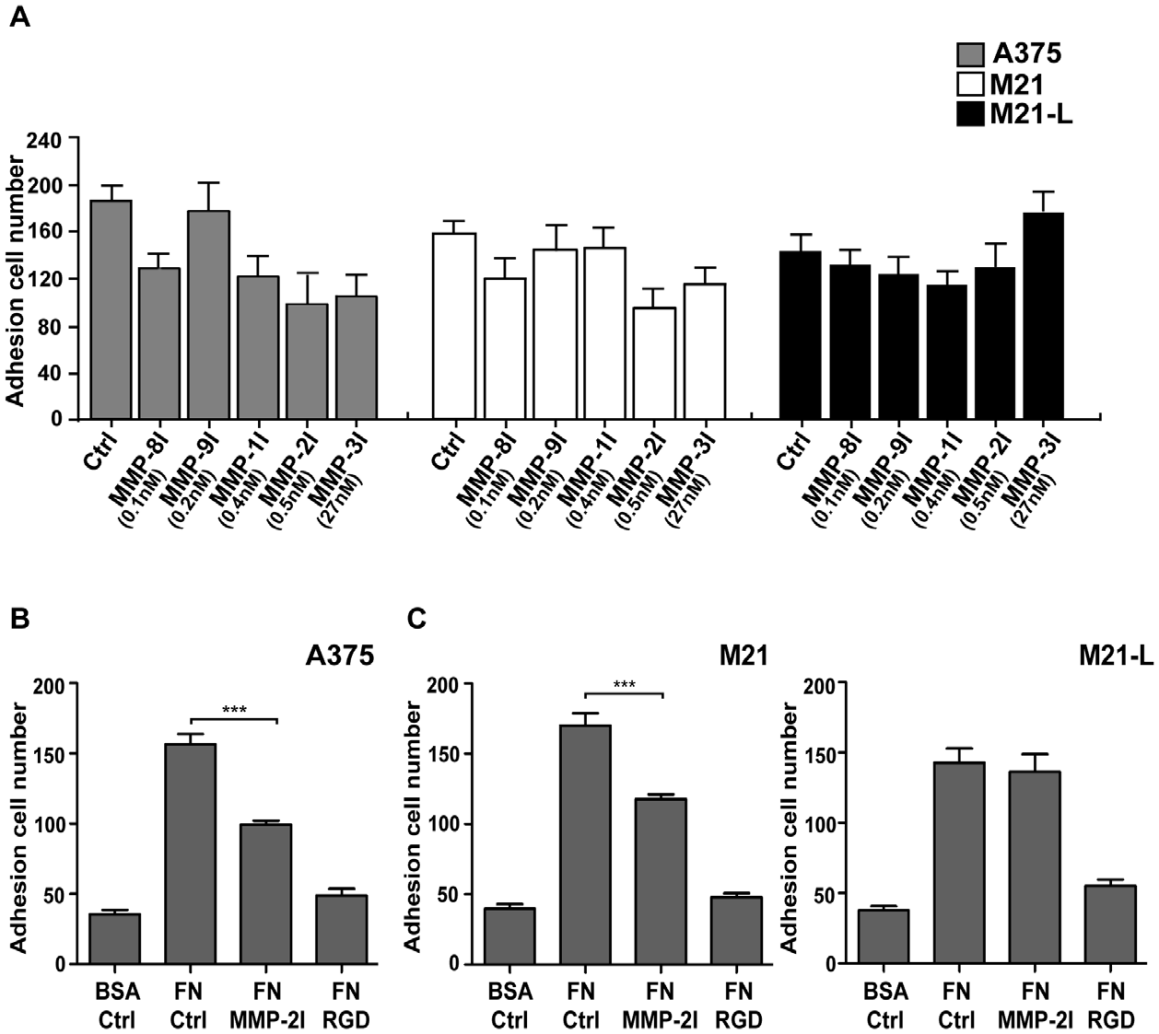
The effect of GM6001 on the adhesion of human melanoma cells. (A) Human melanoma cells, A375, M21 and M21-L, were treated with different concentrations of GM6001, then fluorescently stained and seeded on 48-well plates coated with 10 µg/ml human fibronectin (FN). GM6001 (a broad spectrum hydroxamate MMPs inhibitor) was applied at different concentrations in order to address the role of MMP individuals on the tumor cell adhesion as described in MATERIALS AND METHODS. (B) A375 cells were seeded on human fibronectin (FN) or 0.5% bovine serum albumin (BSA)-coated 48-well plates for 30 min. The cells were treated with or without RGD peptides or 0.5 nM GM6001; (C) M21 and M21-L cells were seeded on human fibronectin (FN) or 0.5% BSA-coated 48-well plates for 30 min. The cells were treated with or without RGD peptides or 0.5 nM GM6001. The fluorescence intensity was measured using a fluorescence spectrophotometer. Statistical difference were determined by comparing the treated group with the normal control by t-test (***, *P*<0.05).

### MMP-2 Activity Regulates αvβ3 Integrin-Mediated the Adhesion of Human A375 Melanoma Cell on Fibronectin

Based on the observation that MMP-2 is recruited to the leading edge of invasive A375 cells before αvβ3 integrin during migration (Figure 2), we hypothesized that MMP-2 may regulate αvβ3 integrin-mediated A375 cell adhesion. A static adhesion assay was employed to detect the effect of MMP-2 on the adhesion of A375 cells mediated by αvβ3 integrin. We first assessed the effects of synthetic inhibitor GM6001 for MMPs (IC_50_ values have been reported as follows: 0.4 nM for MMP-1; 0.5 nM for MMP-2; 27 nM for MMP-3; 0.1 nM for MMP-8 and 0.2 nM for MMP-9) [45]. As shown in Figure 3, we treated the human A375 melanoma cells with different concentration of GM6001, and we found that the cell adhesion was efficiently inhibited by GM6001 at concentration of 0.5 nM, implying an inhibition on MMP-2 as reported [45] (Figure 3A). Higher or lower concentrations of GM6001 did not provide further inhibition. The MMP-2 inhibition (MMP-2I) significantly reduced the adhesion of human A375 cells by approximately 37% when compared with that of control cells (Figure 3B). To determine whether the MMP-2I-reduced adhesion was mediated by αvβ3 integrin, human M21 and M21-L melanoma cells were used in the static adhesion assay (Figure 3C). M21-L cells were selected in the present study because they weakly express αvβ3 integrin and served as a negative control. We found that MMP-2I significantly reduced the adhesion of M21 cells by approximately 30% when compared with that of control cells, but MMP-2I did not induce significant changes in M21-L cells. RGD peptides were used as another control to block integrin-mediated cell-fibronectin interactions. The results demonstrated that MMP-2 activity was essential for αvβ3 integrin-dependent adhesion of human melanoma cells.

**Figure 4:**
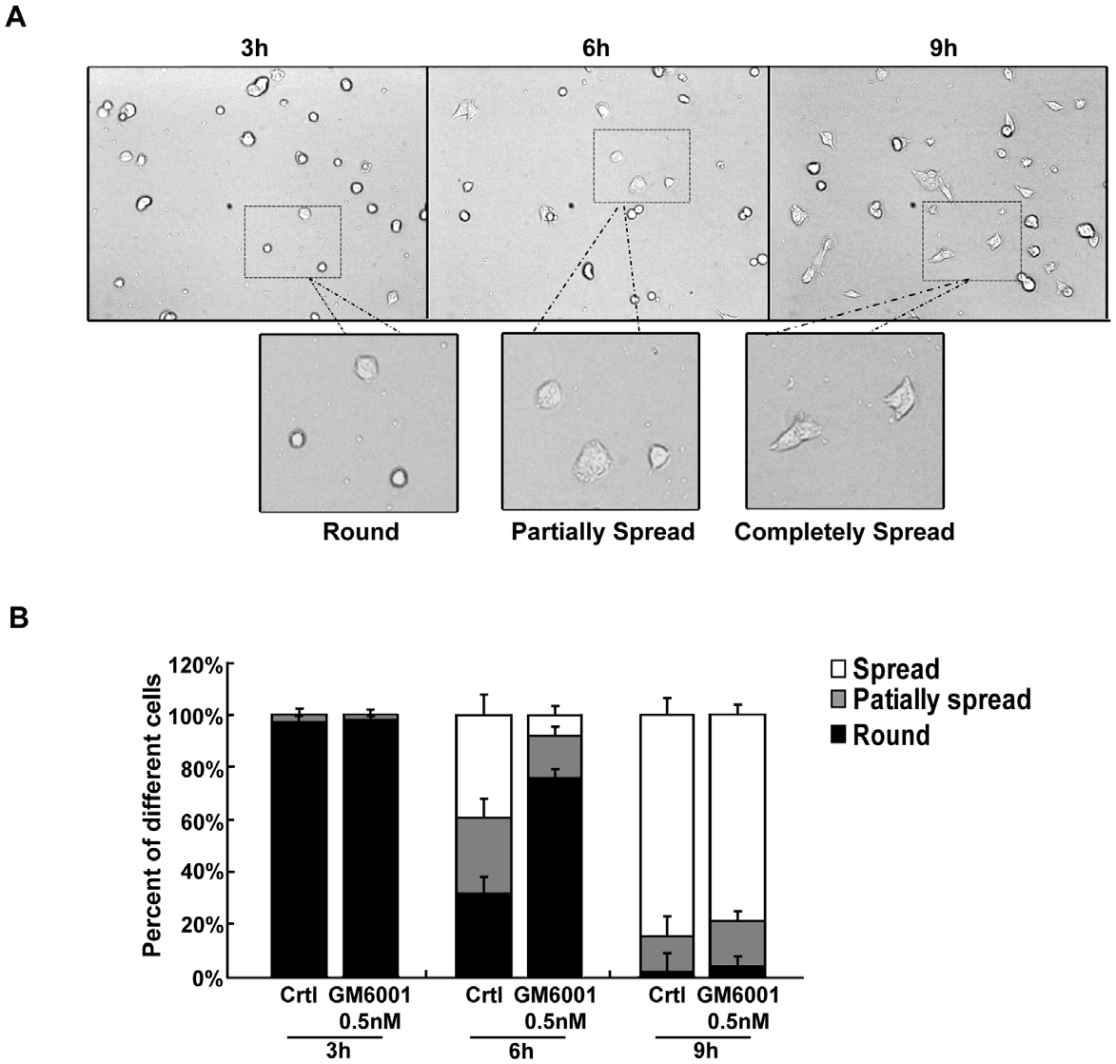
MMP-2 activity affects the spreading of human A375 melanoma cells. (A) The morphology of A375 cell spreading at different time points were designated as round, partially spread and completely spread. (B) Cells were seeded onto coverslips coated with fibronectin, treated with MMP-2I at the concentrations of 0.5 nM at different time points after seeding, and assessed under the microscope. The cells without MMP-2I were defined as control. The differences of cell morphology at 3 h, 6 h and 9 h after seeding were observed and counted (n≥400).

**Figure 5:**
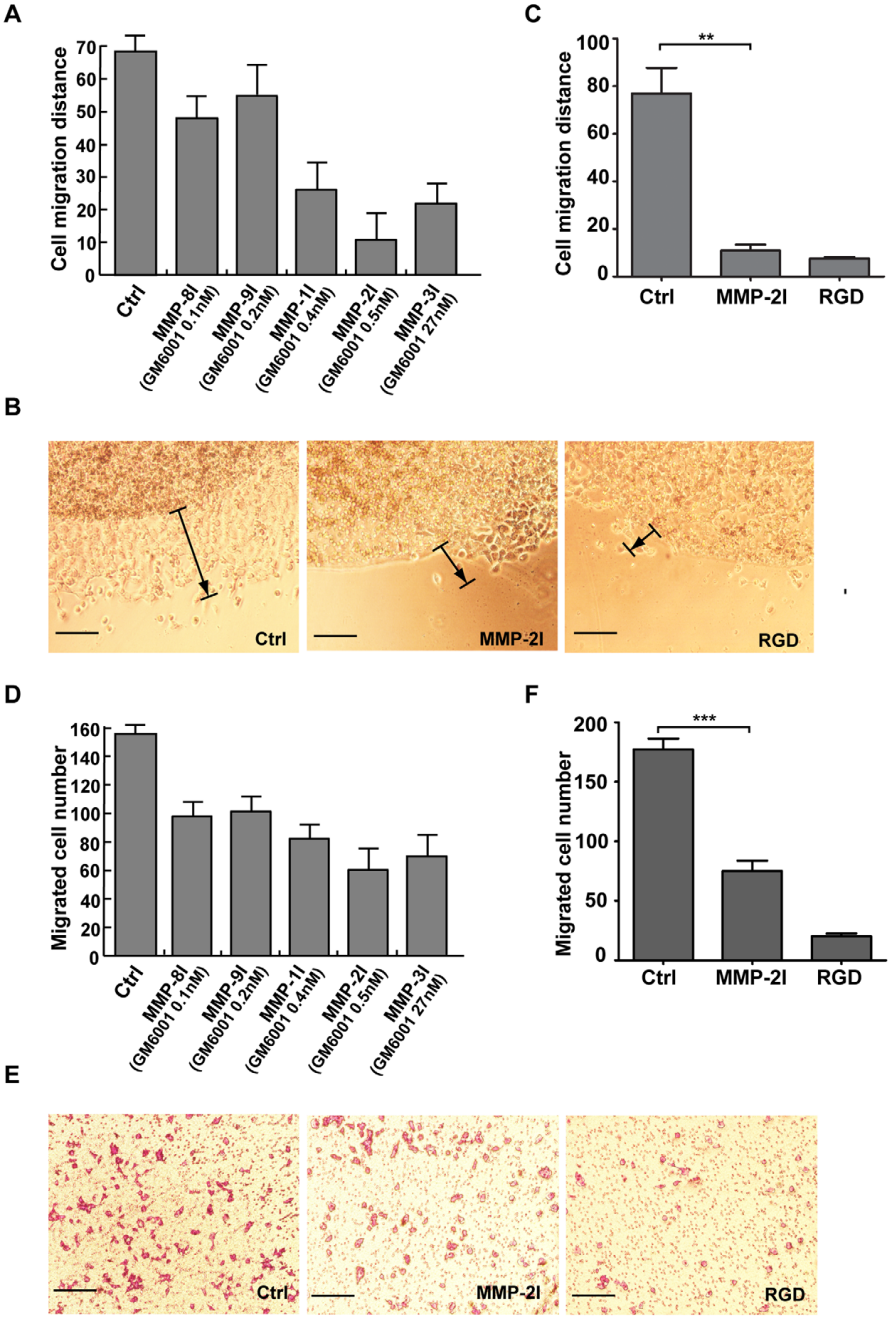
MMP-2 activity affects the migration of human A375 melanoma cells. (A) The effects of GM6001 on the migration of tumor cells in the agarose drop model. GM6001 were applied at different concentrations regarding its function on the different MMP individuals as described in MATERIALS AND METHODS. (B) Photomicrographs showed the A375 cell migration in the agarose drop model 48 h after seeding. The arrows point the direction and distance of cell migration. Cells were untreated (ctrl) or treated with RGD or MMP-2I. Scale bar represents 100 µm. (C) The distance of cell migration was measured. The extent of migrating cells out of the drop = [(total area/drop area)×100] - 100, and the mean values ± SEM were obtained from at least three independent experiments. (**, *P*<0.05). (D) The effects of GM6001 on the tumor cell migration was measured by transwell assay. GM6001 was applied at different concentrations regarding its function on the different MMP individuals as described in MATERIALS AND METHODS. (E) Photomicrographs showed the effects of MMP-2I and RGD on the migration of A375 cell 24 h after seeding in the transwells. Scale bar = 100 µm. (F) The number of migrated cells from different groups was calculated by transwell assay (***, *P*<0.05). Statistical difference was determined by comparing the treated group with the normal control by t-test.

### Inhibition of MMP-2 Activity Affects the Spreading of Human A375 Melanoma Cells

The morphology of migrating cells is an important parameter and reflects the ability of cells in migration. Therefore, we investigated whether MMP-2 activity affects the spreading of human A375 melanoma cells. The cells treated with MMP-2I were seeded onto fibronectin-coated coverslips, and the spreading cells were analyzed and quantified at different time points after seeding (Figure 4). Spreading cells were divided into three groups according to their morphology as follows (Figure 4A): round (the edge of cell is round and smooth), partially spread (the lamellipodia of cell were partially stretched) and completely spread (the lamellipodia of cell were completely stretched). We found almost all of the cells were round at 3 h after seeding (Figure 4B). Six hours later, approximately 39% of cells were completely spread and 29% were partially spread in the untreated group, whereas only 8% of cells were completely spread and 16% were partially spread in the MMP-2I group. However, no significant difference was observed between the groups with or without MMP-2I at 9 h. These data demonstrated that MMP-2 activity affected the spreading of human A375 melanoma cells.

**Figure 6:**
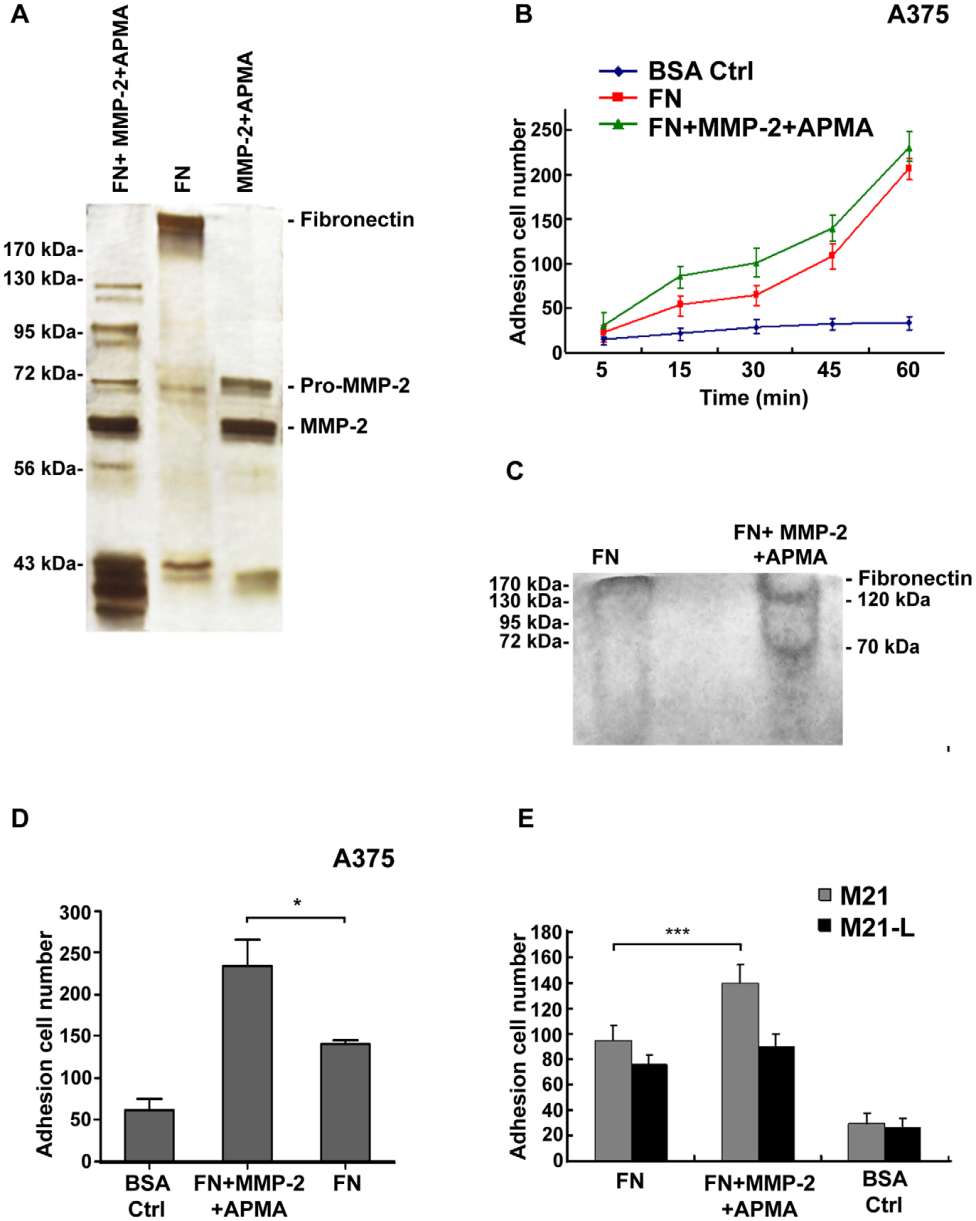
MMP-2 enhances the adhesion of melanoma cells mediated by αvβ3 integrin by cleaving fibronectin. (A) Full-length fibronectin (FN) was incubated with activated MMP-2, and the cleaved fragments (FN + MMP-2+ APMA) were resolved on a 10% Tris-HCl gel and subjected to silver staining. (B) Adhesion assays were performed using human A375 cells incubated in full-length fibronectin (FN)- or MMP-2–cleaved fibronectin (FN+MMP-2+APMA)-coated plates as described in MATERIALS AND METHODS (Cell Adhesion Experiment) at different time points (from 5 to 60 min). (C) Full-length fibronectin (FN) and MMP-2–cleaved fibronectin (FN+MMP-2+APMA) were electrophoresed in a native gel and transferred to nitrocellulose. An adhesion assay was performed using 1.0×10^7^/ml A375 cells incubated on nitrocellulose for 4 h. The membrane was washed and fixed, and the bound cells were stained. (D) The cleaved fibronectin fragments that cells can bind to were retrieved from the native gel, then coated on 24-well flat-bottom plates. An adhesion assay was performed using human A375 melanoma cells incubated in the coated plates for 30 min (*P<0.05). (E) An adhesion assay was performed using human M21 and M21-L melanoma cells incubated in the full-length fibronectin (FN)- or MMP-2–cleaved fibronectin (FN+MMP-2+APMA)-coated plates for 30 min (***P<0.05). Statistical differences were determined by comparing the treated group with the normal control by t-test.

### MMP-2 Activity Regulates αvβ3 Integrin-Mediated Migration of Human A375 Melanoma Cells on Fibronectin

Base on the findings of the static adhesion assay and spreading analysis, we further queried whether MMP-2 activity regulates αvβ3 integrin-mediated migration of human A375 melanoma on fibronectin. In the agarose drop model, A375 cells migrated on fibronectin-coated coverslips, and a linear expansion of corona-like region consisting of migrating cells around the drop was observed over 48 h, and the cells in the migrated area were measured and statistically analyzed (Figure 5). Different concentration of GM6001 was used, and 0.5 nM GM6001 (MMP-2I) was found to efficiently inhibit cell migration (Figure 5A). The extension of the corona-like area of MMP-2I treated cells was considerably diminished compared with that of untreated control cells, and RGD peptide dramatically blocked the outward dissemination of cells from the drop (Figure 5B). We measured the drop area and total area (area of the drop + area of migrating cells). Cell migration was expressed as the extent of migrating cells out of the drop = [(total area/drop area)×100] - 100. As shown in Figure 5C, RGD peptides reduced the cell migration by more than 90%, and the extension of the area of MMP-2I treated group was approximately 85% less than that of the control group, indicating that MMP-2 activity was required for the A375 cell migration. To further understand the role of MMP-2 activity in A375 cell migration, a transwell assay was performed. Different concentrations of GM6001 were also applied, and MMP-2I (0.5 nM GM6001) was found to efficiently inhibit cell migration (Figure 5D). MMP-2I treatment dramatically inhibited cell migration through the fibronectin-coated polycarbonate membrane compared with the untreated group (Figure 5E). The number of migrated cell in the MMP-2I treated group was approximately 57% lower than that of the control group, and RGD peptides also reduced the cell migration nearly by 88% (Figure 5F). The results indicated that MMP-2 activity played a critical role in A375 cell migration.

### MMP-2 Enhances the αvβ3 Integrin-Mediated Adhesion of Human A375 Melanoma Cells by Cleaving Fibronectin

MMP-2 cleaves various ECM proteins to implement different regulatory functions [17], [47]. We investigated whether MMP-2 cleaved fibronectin to enhance the adhesion of melanoma cells mediated by αvβ3 integrin. Recombinant human MMP-2 was activated using APMA and incubated with full-length fibronectin (Figure 6A). The mixture was resolved by SDS-PAGE. Silver staining of the gel revealed several fibronectin fragments. To test whether the cleavage of fibronectin was functionally significant, cell adhesion assays were conducted on the cleaved fibronectin fragments at different time points (Figure 6B). As expected, the difference was not significant between MMP-2-cleaved fibronectin and control groups at 5 min. Thirty minutes later, approximately more than 36% A375 cells adhered to the culture wells coated with MMP-2–cleaved fibronectin compared with those coated with full-length fibronectin alone. The ratio was about 23% at 5 min, 36% at 15 min, 36% at 30 min, 22% at 45 min and 11% at 60 min respectively. It suggested that MMP-2 enhance the adhesion of A375 melanoma cells by cleaving fibronectin obviously at 30 min. This result was further confirmed by a nitrocellulose filter attachment assay (Figure 6C), in which full-length or MMP-2-cleaved fibronectin was resolved on a native Tris-HCl gel and transferred to nitrocellulose, and the cells that were bound to the filter were detected by amino black cell staining. We found that A375 cells preferentially bound to 120-kDa and 70-kDa bands, which are known as MMP-2–cleaved fibronectin fragments. To test whether the MMP-2-cleaved 120-kDa and 70-kDa bands are functional for enhancing the adhesion of melanoma mediated by αvβ3 integrin, we retrieved the fragments from the native gel and coated them on 24-well flat-bottom plates (Figure 6D). An adhesion assay was performed using human A375 melanoma cells, and the results showed that approximately more than 39% of cells adhered to the wells coated with fragments compared with those coated with retrieved full-length fibronectin. To further certify the participation of αvβ3 integrin in the process showed above, adhesion assay were performed again using human M21 and M21-L melanoma cells (Figure 6E). Strikingly, approximately more than 34% of adherent cells were detected with the M21 cells, whereas, no obvious changes were detected with the M21-L cells.These results suggested that αvβ3 integrin is indispensible for the cell adhesion facilitated by MMP2-mediated fibronectin cleavage, and that MMP-2–cleaved fibronectin promote the adhesion of tumor cells.

## Discussion

Invasive migration of tumor cells in the basement membrane is dependent on cell adhesion and migration as well as proteolysis of the extracellular matrix, and is the initial step of tumor metastasis. The proteolytic activation of MMP-2 and the adhesive function of αvβ3 integrin are involved in the process [10]. MMP-2 directly binds to αvβ3 integrin via the C-terminus of active MMP-2 [41]. Previous studies have mainly focused on a αvβ3-MMP-2 mechanism to demonstrate how αvβ3 integrin regulates MMP-2. However, it is not clear whether MMP-2 reciprocally contributes to αvβ3 integrin regulation. In present work, we demonstrated that MMP-2 may promote the recruitment of αvβ3 integrin and regulate αvβ3 integrin-mediated tumor cell migration.

We investigated the spatiotemporal relationship between MMP-2 and αvβ3 integrin expression. We showed that MMP-2 was detected on the surface of tumor cells after the cells initially attached to matrix, nevertheless, the signal of co-localization between MMP-2 and αvβ3 integrin was barely detectable (Figure 1C). Once the cells were completely spread, both MMP-2 and αvβ3 integrin were strongly expressed. These results imply that MMP-2 is recruited on the surface of tumor cells before αvβ3 integrin. To further confirm this observation, we performed an agarose drop assay with immunofluorescence analysis. After the tumor cells were resuspended in an agarose drop, the distribution of MMP-2 and αvβ3 integrin was examined from 1 h to 12 h. Cells were permitted to adhere to coverslips for 1 h after seeding. We found MMP-2 displayed a cluster-like signal, which was enriched at the leading edge of the cells. However, the expression of αvβ3 integrin was evenly distributed on the surface of the cells. When an increasing number of cells were adhered to the coverslips at 3 h or 7 h after seeding, partial co-localization of MMP-2 and αvβ3 integrin was observed (Figure 2). Based on these findings, we speculated that MMP-2 may regulate αvβ3 integrin. To verify this hypothesis, we first tested whether MMP-2 activity was involved in tumor cell adhesion. Our static adhesion assay showed that the inhibition of MMP-2 impaired the adhesion of tumor cells (Figure 3A), suggesting that MMP-2 activity may regulate the adhesion of tumor cells. To obtain some mechanistic insights, we examined whether the MMP-2 regulated adhesion of tumor cells requires αvβ3 integrin. We employed human M21 (wild-type of αvβ3) and M21-L (αvβ3 defective) melanoma cells and found that tumor cell adhesion was αvβ3 integrin-dependent (Figure 3B). Next, we detected the effect of MMP-2 activity on cell spreading. Morphometric analysis of the tumor cells revealed the differences in the rate of cell spreading after MMP-2 inhibition (Figure 4 B), indicating that MMP-2 activity affected the spreading of tumor cells. Furthermore, we showed that MMP-2 inhibition strongly limited the tumor cell migration both in the agarose drop model and the transwell assay (Figure 5). Collectively, these results demonstrated that MMP-2 regulates αvβ3 integrin-dependent tumor cell migration.

MMP-2 can cleave various ECM proteins [17]. However, whether these cleavage products affect αvβ3 integrin-mediated tumor cell migration is not well understood. We hypothesize that MMP-2 may cleave ECM and facilitate the binding of αvβ3 integrin to ECM. Fibronectin is a substrate of MMP-2 and a ligand of αvβ3 integrin. We investigated the possibility that MMP-2 cleaves fibronectin into shorter fragments to facilitate the interaction between αvβ3 integrein and cleaved fibronectin. Indeed, we found that wild-type αvβ3 integrin-expressed cells preferentially attached to the cleaved shorter fibronectin fragments (Figure 6). In contrast, αvβ3 integrin-mutant cells showed similar adhesive abilities before and after fibronectin digestion. We propose the following mechanism for the initial steps of invasion based on tumor cell adhesion and migration. Active MMP-2 is first recruited to the leading edge of invasive tumor cells and cleaves fibronectin into shorter fibronectin products. The fibronectin fragments promote αvβ3 integrin recruitment to the area of cleaved fibronectin products to facilitate tumor cell adhesion and migration. Collectively, αvβ3 integrin-mediated adhesion and migration of tumor cells is regulated, at least in part, by MMP-2 via fibronectin cleavage.

Additionally, as we were conscious of the contribution of another integrin, β1 integrin, in the adhesion of tumor cells to fibronectin, we detected the expression of β1 on the selected cells and its effects on the initial cell adhesion on fibronectin. FACS experiments showed that all the selected cells highly expressed β1 integrin, and β1 integrin blocking with antibody affected the adhesion of the tumor cells to fibronectin. This may explain why melanoma cell adhesion to fibronectin is reduced in the αvβ3-deficient cells, but not equal to BSA control. Immunofluorescence experiments showed that in early phases of tumor cell adhesion, β1 integrin may form clusters earlier than αvβ3 integrin, suggesting that β1 integrin may play adhesion role earlier than αvβ3 integrin. These results implicated β1 integrin also play an important role in the initial adhesion of tumor cells (Figure S1). We also detected another human tumor cells (human DU145 prostatic cancer cell) with several assays as above. The results showed a similar tendency (Figure S2, S3), which imply that MMP-2 and αvβ3 integrin may regulate each other in different cells.

## Supporting Information

Figure S1 **The effects of β1 integrin expression on the adhesion of human melanoma cells (A375, M21 and M21-L).** (A) The expression of β1 integrin on human melanoma cell surface was evaluated by flow cytometric analysis using anti-β1 antibodies. Isotype IgG was used as negative control. (B) Human melanoma cells were fluorescently stained and seeded on 48-well plates coated with 10 µg/ml human fibronectin (FN) or 0.5% BSA. The cells were treated with β1 antibody, RGD peptides or MMP-2I. (C) A375 cells were seeded onto coverslips coated with human fibronectin for 1 h, then labeled with anti-αvβ3 or anti-β1 antibodies respectively. Scale bar = 10 µm.
(TIF)

Figure S2 **Expression and interaction of MMP-2 and αvβ3 integrin in tumor cells.** (A) Gelatin zymography of three kinds of tumor cells. Lane 1 & lane 2: human A375 melanoma cells; lane 3 & lane 4: human M21 melanoma cells; lane 5 & lane 6: human DU145 prostatic cancer cells. All these cells expressed gelatinases. (B) Flow cytometric analysis of αvβ3 integrin and MMP-2 expression on the DU145 cell surface. The expressions were evaluated using anti-αvβ3 and anti-MMP-2 antibodies, and isotype IgG was used as negative control. (C) Coimmunoprecipitation assay of αvβ3 integrin and MMP-2 in tumor cells. Upper panel, MMP-2 was in the immunoprecipitated complex of αvβ3 integrin; Lower panel, αvβ3 integrin was in the immunoprecipitated complex of MMP-2. (D) DU145 cells were suspended in PBS and labeled with anti-αvβ3 (red) and anti-MMP-2 (green) antibodies. Scale bar = 20 µm.
(TIF)

Figure S3 **The effect of MMP-2 activity on the adhesion and migration of human DU145 prostatic cancer cells.** (A) The effects of GM6001 on DU145 cell adhesion were analyzed in different concentration regarding its function on the different MMP individuals as described in MATERIALS AND METHODS. (B) DU145 cells were fluorescently stained and seeded on 48-well plates coated with 10 µg/ml human fibronectin (FN) or BSA as control. The cells were treated using RGD peptides or MMP-2I. The fluorescence intensity was measured using a fluorescence spetrophotometer. Statistical difference were determined by comparing treated group with the normal control by t-test (**, P<0.005). (C) The photomicrographs of DU145 cells in the agarose drop model 48 h after seeding. The arrows point the direction and distance of cell migration. Scale bar represents 100 µm. (D) Photomicrograph of the effects of MMP-2I on DU145 cell migration 24 after seeding in the transwell. Scale bar = 100 µm.
(TIF)
