# Supplementary figures and images for: Matrix metalloproteinase-2 Promotes αvβ3 Integrin-Mediated Adhesion and Migration of Human Melanoma Cells by Cleaving Fibronectin
Source: PLoS One. 2012 Jul 27;7(7):e41591. doi: 10.1371/journal.pone.0041591 (PMC3407216; doi:10.1371/journal.pone.0041591)

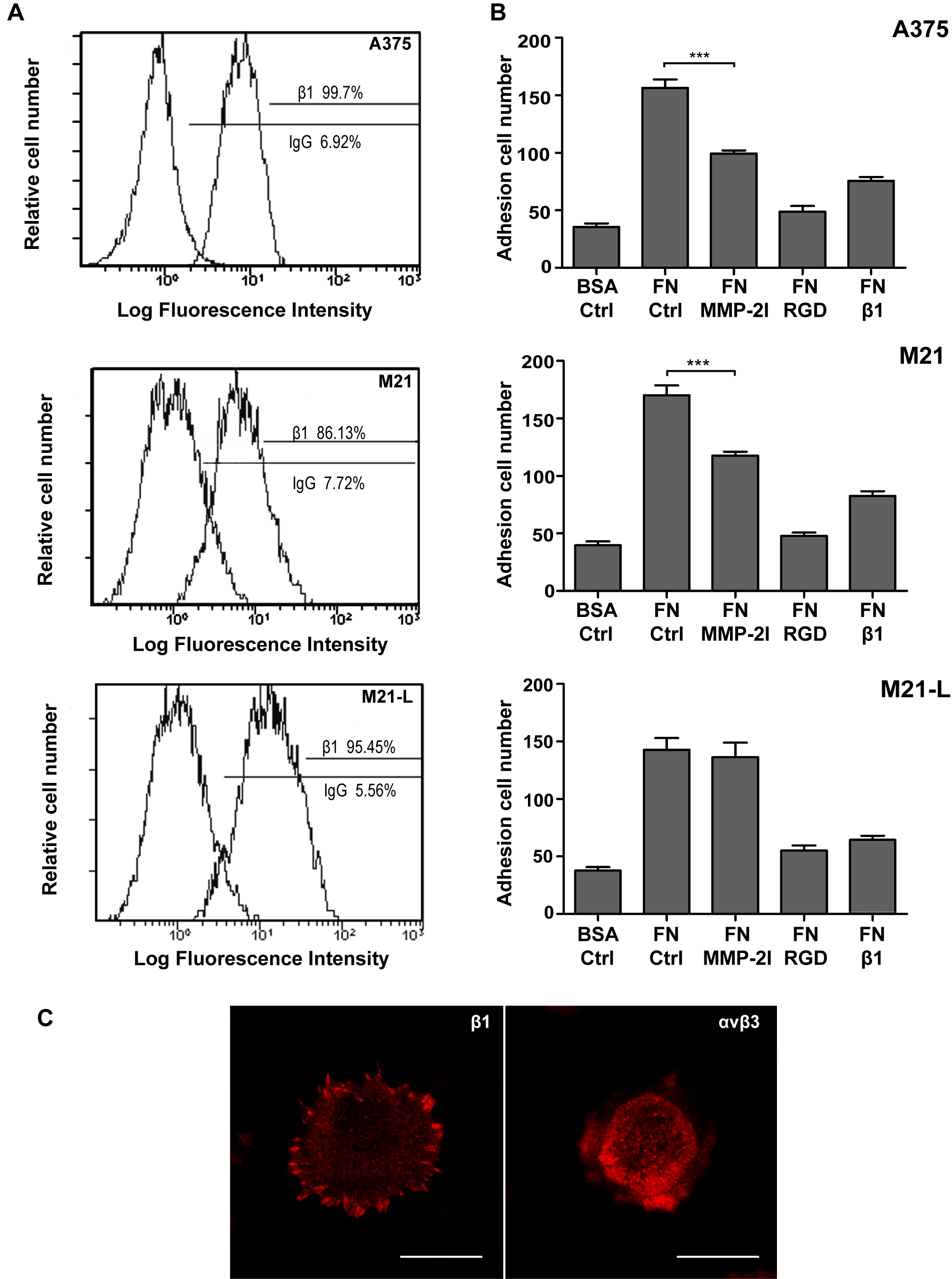

Supplement: Figure S1 — The effects of β1 integrin expression on the adhesion of human melanoma cells (A375, M21 and M21-L). (A) The expression of β1 integrin on human melanoma cell surface was evaluated by flow cytometric analysis using anti-β1 antibodies. Isotype IgG was used as negative control. (B) Human melanoma cells were fluorescently stained and seeded on 48-well plates coated with 10 µg/ml human fibronectin (FN) or 0.5% BSA. The cells were treated with β1 antibody, RGD peptides or MMP-2I. (C) A375 cells were seeded onto coverslips coated with human fibronectin for 1 h, then labeled with anti-αvβ3 or anti-β1 antibodies respectively. Scale bar = 10 µm. (TIF) [file pone.0041591.s001.tif]

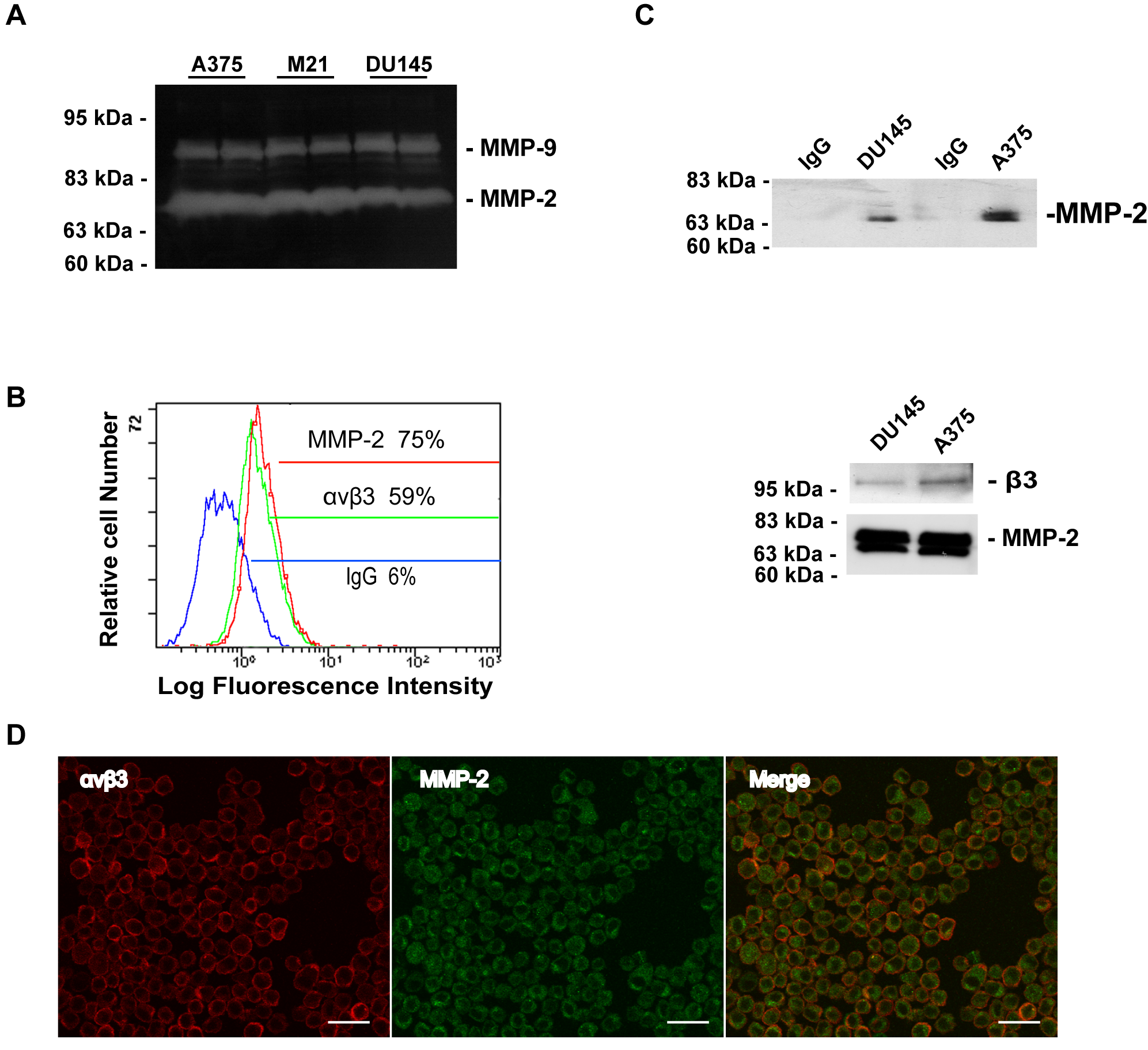

Supplement: Figure S2 — Expression and interaction of MMP-2 and αvβ3 integrin in tumor cells. (A) Gelatin zymography of three kinds of tumor cells. Lane 1 & lane 2: human A375 melanoma cells; lane 3 & lane 4: human M21 melanoma cells; lane 5 & lane 6: human DU145 prostatic cancer cells. All these cells expressed gelatinases. (B) Flow cytometric analysis of αvβ3 integrin and MMP-2 expression on the DU145 cell surface. The expressions were evaluated using anti-αvβ3 and anti-MMP-2 antibodies, and isotype IgG was used as negative control. (C) Coimmunoprecipitation assay of αvβ3 integrin and MMP-2 in tumor cells. Upper panel, MMP-2 was in the immunoprecipitated complex of αvβ3 integrin; Lower panel, αvβ3 integrin was in the immunoprecipitated complex of MMP-2. (D) DU145 cells were suspended in PBS and labeled with anti-αvβ3 (red) and anti-MMP-2 (green) antibodies. Scale bar = 20 µm. (TIF) [file pone.0041591.s002.tif]

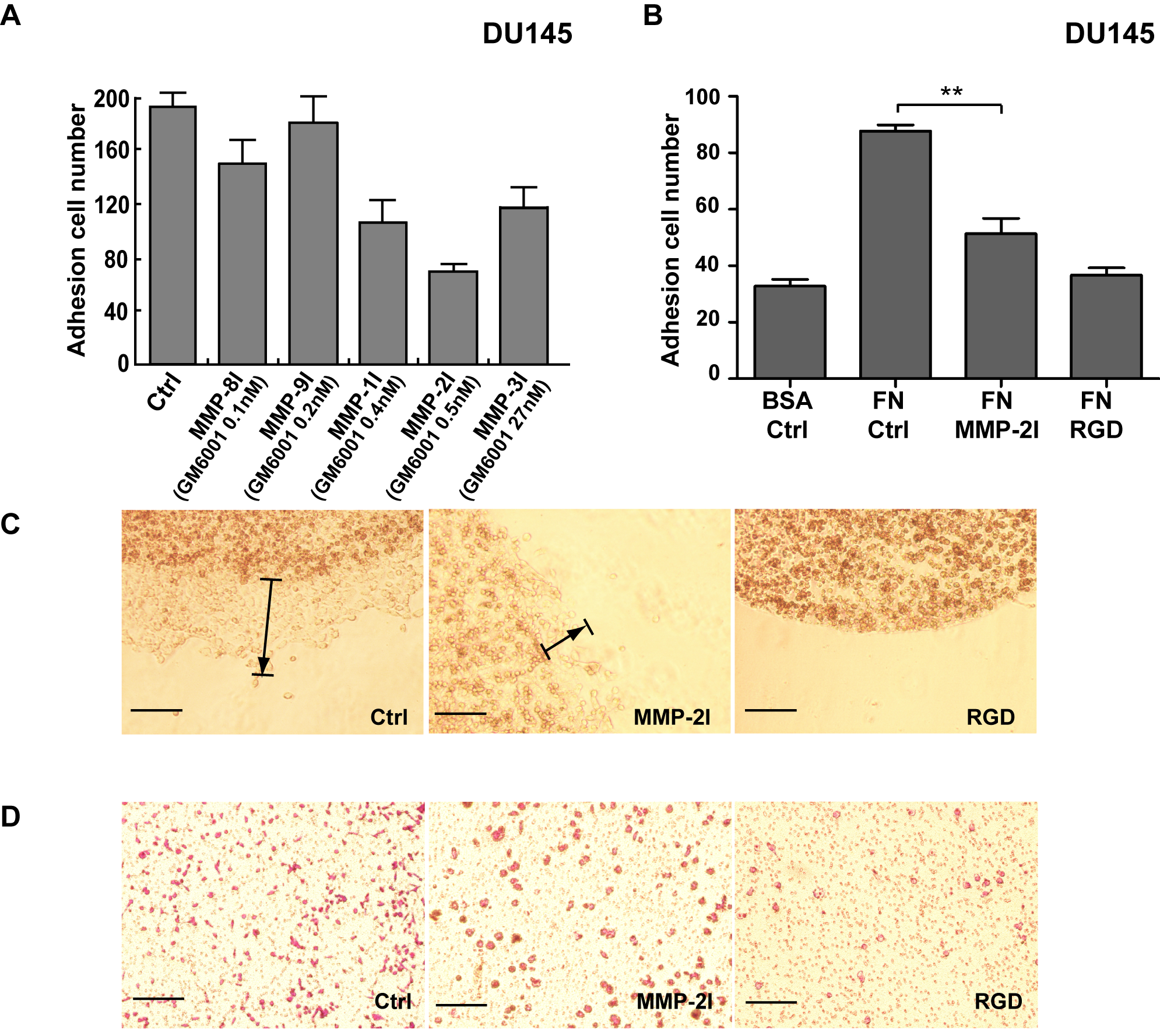

Supplement: Figure S3 — The effect of MMP-2 activity on the adhesion and migration of human DU145 prostatic cancer cells. (A) The effects of GM6001 on DU145 cell adhesion were analyzed in different concentration regarding its function on the different MMP individuals as described in MATERIALS AND METHODS. (B) DU145 cells were fluorescently stained and seeded on 48-well plates coated with 10 µg/ml human fibronectin (FN) or BSA as control. The cells were treated using RGD peptides or MMP-2I. The fluorescence intensity was measured using a fluorescence spetrophotometer. Statistical difference were determined by comparing treated group with the normal control by t-test (**, P<0.005). (C) The photomicrographs of DU145 cells in the agarose drop model 48 h after seeding. The arrows point the direction and distance of cell migration. Scale bar represents 100 µm. (D) Photomicrograph of the effects of MMP-2I on DU145 cell migration 24 after seeding in the transwell. Scale bar = 100 µm. (TIF) [file pone.0041591.s003.tif]
